# Differential associations of childhood abuse and neglect with neural responses to social reward and punishment in adults with anxiety or depression

**DOI:** 10.1038/s41398-026-03881-2

**Published:** 2026-02-06

**Authors:** Isabella G. Spaulding, Murray B. Stein, Charles T. Taylor

**Affiliations:** 1 San Diego State University/University of California San Diego, Joint Doctoral Program in Clinical Psychology, San Diego, CA USA; 2 Department of Psychiatry, University of California San Diego, San Diego, CA USA; 3 School of Public Health, University of California San Diego, San Diego, CA USA

**Keywords:** Neuroscience, Psychology

## Abstract

Childhood adversity increases risk for impaired social functioning later in life; however, neural pathways delineating this association are poorly understood. Different types of adverse interpersonal experiences (i.e., abuse, neglect) may impact neural pathways distinctly, resulting in unique consequences on social motivation and behavior in adulthood. Here, we investigated neural responses during the anticipation of social reward and punishment and their associations with childhood abuse and neglect in adults with a depressive and/or anxiety disorder and social impairment. Participants (*N* = 57) completed an fMRI social incentive delay task. In region-of-interest analyses, we examined activation in striatal regions when participants anticipated receiving potential social reward or avoiding social punishment in relation to self-reported childhood abuse and neglect. Individuals endorsing greater neglect during childhood demonstrated increased activation in the caudate (β = 0.359, *p* = 0.006) and putamen (β = 0.454, *p* < 0.001) during anticipation of social reward, while participants reporting greater abuse during childhood showed decreased activation in the same regions (β = −0.314, *p* = 0.024 and β = −0.341, *p* = 0.014, respectively). When both types of adversity were included in the same model, only neglect remained a significant predictor of putamen activation during social reward anticipation (β = 0.402, *p* = 0.009). No significant associations were observed between childhood trauma and activation during anticipation of social punishment. Findings reveal differential associations of childhood abuse and neglect with dorsal striatum activation during social reward anticipation in adults with anxiety and depression. Treatments targeting aberrant social reward processing may benefit patients who have experienced significant childhood adversity, and different approaches may be needed based on the type(s) of early adversity experienced.

## Introduction

Social disconnection is common in anxiety and depression [1, 2] and is associated with greater symptom severity and lower rates of recovery [3, 4]. First-line treatments do not sufficiently address social disconnection and social impairments often persist after other negative affective symptoms remit [2]. A better mechanistic understanding of social disconnection in depression and anxiety is needed for improved treatment approaches and personalization. Notably, childhood adversity increases vulnerability for both social impairment and later psychopathology. Theoretical frameworks from translational and clinical neuroscience propose early adverse experiences affect brain responses to reward, resulting in long-term reward processing deficits often linked with depression [5–7] and anxiety disorders [8]. While many studies have explored relationships between nonsocial reward processing and early adverse experiences, relationships to social reward processing remain understudied, including both the presence of positive social outcomes (e.g., acceptance) as well as the absence of negative social outcomes (e.g., criticism). Here, we examined associations among social reward processing and different types of early adversity in an adult sample of individuals with social functioning impairments seeking care for anxiety and/or depression.

Early adverse experiences are often interpersonal in nature, frequently occurring in the context of some of our earliest and most important social relationships. For example, early adversity can be the result of a direct social encounter where a child experiences harmful and threatening behavior (e.g., *abuse*). Alternatively, adverse experiences of *neglect* can arise from indirect social encounters, where trusted caregivers do not provide expected emotional or material support. Throughout development, these experiences shape expectancies for social encounters and facilitate learned behaviors [9, 10]. Social schema and behaviors may then generalize to new situations and affect sociality later in life. For instance, extant work has linked childhood adversity with impaired social functioning in adulthood, including greater interpersonal problems [11], lower social functioning [12], less social support [13, 14], and fewer friends [15]. Importantly, adverse social experiences may manifest long-term maladaptive alterations in brain function that impact broader systems of incentive processing, such as attention towards meaningful stimuli or approach behavior [6, 16].

The striatum is centrally involved in regulating incentive processing-- the ventral striatum (VS) is implicated in reward valuation and approach motivation, while dorsal striatal regions link reward incentives to action [17]. Existing work exploring associations between striatal activation and early adversity has often taken a cumulative perspective of adversity (e.g., summing types of adversities experienced). Most prior studies have found cumulative adversity is associated with blunted striatal responses during reward anticipation in youth [5, 18, 19] and adults [20–22], though several studies did not observe a relationship between the two [23–25]. Critically, the vast majority of this work focused on non-social rewards (e.g., money, points), which may not capture deficits in social reward processing that may underlie symptoms such as social anhedonia and withdrawal [6, 8, 26]. The few existing studies examining social reward report mixed findings and primarily explore reward responsiveness (cf. anticipation). In previously institutionalized youth, Goff et al. [27] observed less activation in the VS to happy faces. However, in young adults, greater early life stress was associated with increased activation in reward regions (insula, pallidum, substantia nigra) to receipt of verbal versus monetary reward [28], and adolescent girls with high levels of family adversity had strengthened functional connectivity between the VS and other reward regions when receiving positive peer feedback [16]-- both findings suggesting elevated sensitivity to receipt of social reward.

Cumulative approaches to early adversity may obscure individual differences in early adverse experiences and how these experiences may uniquely impact social reward processes. One conceptual model of adversity proposes that two different types of adversity, threat (presence of harmful, unexpected stimuli; e.g., physical, sexual, or emotional abuse) and deprivation (absence of positive, expected stimuli, e.g., physical or emotional neglect), impact neurobiological mechanisms in distinct ways and are associated with unique developmental consequences [29–31]. In the context of social reward processing, different types of childhood adversity may be associated with different mechanistic pathways toward later social disconnection. For example, frequent early experiences of abuse, such as those that signal the possibility of social punishment, may shape neural pathways and corresponding motivation and behavior in different ways compared to the presence of neglect, which may reflect fewer opportunities for social reward. Better understanding of these pathways is important to ultimately identifying whether different treatment targets or approaches may be needed to address social impairments.

Limited work has explored how childhood abuse or neglect impacts social reward processing, particularly anticipatory phases, and few studies have considered both types of adversity within the same sample. Of the studies examining childhood neglect, one study observed blunted striatal response to reward receipt (i.e., happy faces) in adolescents with greater social deprivation [32]. Another study observed no associations between neglect and VS activation to happy faces in young adults, however, greater neglect was associated with increased orbitofrontal cortex activation, a region involved in determining reward value [33]. In regards to childhood abuse, heightened activation was observed in the VS and putamen to images of positive social scenes in adolescents and young adults [34], whereas a study using similar stimuli reported no differences between abuse-exposed and non-exposed youth and young adults [35]. Only one study [36] explored anticipatory phases of social reward processing in relation to childhood abuse and neglect in adults with posttraumatic stress disorder, depression, somatic symptom disorder, and healthy volunteers. They examined multiple forms of adversity (i.e., maternal and paternal neglect, abuse, antipathy, overall sexual abuse) and observed reduced VS activation only in individuals reporting greater maternal antipathy (including elements of maternal neglect and abuse). However, they separately analyzed paternal and maternal abuse and neglect, which may have constrained the study’s ability to detect broader adversity-related neural associations and does not reflect adversity experienced in other social contexts. Overall, these conflicting findings are unsurprising given heterogeneity across samples, including stages of development, adversities of interest, presence or absence of psychiatric symptoms, and reward paradigms.

Early adversity may also impact how one responds to the absence of negative social outcomes, such as the opportunity to avoid social punishment. In tasks where participants can avoid negative stimuli, such as incentive delay tasks, avoiding punishment may also be particularly rewarding. A meta-analysis on the social incentive delay task (SID), where participants can avoid threatening faces, observed similar striatal activation during the anticipation of receiving reward and avoiding punishment [37]. Though speculative, this avoidance may be particularly rewarding for individuals with early adversity, who may be hypersensitive to social threats. While some studies have examined associations between childhood adversity and receipt of social punishment [35, 38, 39] or anticipating monetary loss [23, 25, 40], to our knowledge, no studies have examined associations between childhood adversity and striatal activation when anticipating avoiding social punishment.

Important questions regarding the relationship between childhood adversity and the neural mechanisms of social reward remain. First, prior research examining the relationship between childhood adversity and *social* reward processing is scant, despite its arguably more direct connection to social functioning impairments (e.g., anhedonia, social avoidance) than nonsocial reward. The limited studies examining social reward processing primarily considered activation during passive viewing of positive faces or social scenes, rather than tasks that probe effortful approach behaviors to receive social reward. Exploring anticipation phases in tasks requiring effort may better capture the neural mechanisms underlying social approach and motivation, a stage of reward processing implicated in depression and anxiety [41]. Second, extant literature has mostly included youth and young adults, and it is unclear whether findings reflect specific periods in which reward processing is still developing, or whether these aberrant responses remain throughout adulthood. Third, prior work suggests abuse and neglect may be associated with differential neurobiological and clinical presentations, yet only one prior study has evaluated separate effects of childhood abuse and neglect on social reward processing.

Here, we examined whether different types of early adversity (i.e., abuse, neglect) predicted neural activation during anticipation of social reward or avoidance of social punishment in a priori selected regions implicated in reward processing (i.e., ventral and dorsal striatum) in a transdiagnostic sample of adults with internalizing disorders characterized by social disconnection. Analyses focused on striatal regions as these regions play a central role in social reward valuation and approach behaviors [42, 43], processes essential for connecting with others [44]. We hypothesized that abuse and neglect would predict striatal activation during the anticipation of social reward, however, given mixed findings from prior literature, we did not hypothesize a direction for these associations. Further, as no studies to our knowledge have evaluated activation in reward regions during the anticipation of avoiding social punishment and associations with childhood adversity, we conducted exploratory analyses examining these relationships.

## Methods and materials

### Participants

The sample included 57 participants ages 18–55 (inclusive) enrolled in a randomized clinical trial for individuals with clinically elevated levels of depression or anxiety (ClinicalTrials.gov Identifier: NCT03196544), as indicated by a score of 8 or higher on the Overall Anxiety Severity and Impairment Scale (OASIS) [45] or a 10 or higher on the Patient Health Questionnaire (PHQ-9) [46]. Additional inclusion criteria of the clinical trial required participants to demonstrate social disconnection (Social Connectedness Scale Revised (SCSR) score <90) [47] and moderate or greater social impairment (Sheehan Disability Scale-- social domain score $\ge$5) [48]. Outcomes from this clinical trial have been previously reported [49]. See Supplement for exclusion criteria.

Participants were recruited through primary care clinics and advertisements in online and community settings. Diagnostic interviews to determine clinical diagnoses and evaluate exclusion criteria were conducted using the Mini International Neuropsychiatric Interview for DSM-5 (MINI; version 7.0.2) [50]. See Table 1 for participant demographics and clinical characteristics.

**Table 1:** Participant demographic and clinical characteristics.

Demographic Variable	
Age	30.0 (9.5)
Gender Identity	
Female	39 (68.4)
Male	17 (29.8)
Other	1 (1.8)
Race	
Asian	15 (26.3)
Black	1 (1.8)
More than one race	2 (3.5)
Pacific Islander	1 (1.8)
Unknown/Declined to respond	1 (1.8)
White	37 (64.9)
Hispanic Ethnicity	12 (21.1)
PHQ-9	12.3 (4.9)
OASIS	10.7 (3.0)
CTQ	54.8 (8.9)
CTQ neglect subscore	29.1 (5.7)
CTQ abuse subscore	25.7 (10.9)
Diagnoses^a^	
Major depressive disorder	45 (78.9)
Social anxiety disorder	38 (66.7)
Generalized anxiety disorder	30 (52.6)
Posttraumatic stress disorder	5 (8.8)
Panic disorder	6 (10.5)
Agoraphobia	8 (14.0)
Obsessive-compulsive disorder	4 (7.0)
Eating disorder	4 (7.0)
Mild cannabis use disorder	5 (8.8)
Mild alcohol use disorder	7 (12.3)

Values are presented as mean (SD) or *n* (%).

*OASIS* Overall Anxiety Severity and Impairment Scale, *PHQ-9* Patient Health Questionnaire, *CTQ* Childhood Trauma Questionnaire.

^a^percentages sum to >100% given high comorbidity across sample.

### Procedure

All study procedures involving human participants were performed in accordance with ethical standards of the University of California San Diego Human Research Protection Program and with the Code of Ethics of the World Medical Association (Declaration of Helsinki). All participants provided written informed consent before beginning participation in the study. After completing an initial eligibility appointment, participants completed self-report measures and underwent functional magnetic resonance imaging in a separate visit.

### Social incentive delay task

At baseline, all participants underwent fMRI and completed the social incentive delay (SID) task, a paradigm designed to elicit neural responses to the anticipation and receipt of social reward (e.g., viewing a smiling face) [51]. The SID has been shown to reliably activate reward processing neurocircuitry, including striatal regions [37]. In the SID task, participants were presented with opportunities to gain social reward or avoid social punishment through an on-time response via button-press to a target symbol that was preceded by a cue shape indicating the level of potential reward or punishment (i.e., neutral, low, high). Social reward and punishment feedback were presented via facial expressions of smiling or angry faces, respectively, with varying intensity of expression associated with the level of potential incentive. See the Supplement for a full description and visual depiction of the task.

### Measures

#### Childhood trauma

The Childhood Trauma Questionnaire (CTQ) [52] was used to measure experiences of abuse and neglect in childhood and adolescence. The CTQ demonstrates strong test-retest reliability [53] and convergent and discriminant validity when compared to clinician-assessed reports of maltreatment [52]. The CTQ is a 28-item questionnaire that assesses maltreatment experiences including physical, sexual, and emotional abuse, and physical and emotional neglect. Severity of each item is assessed using a 5-point Likert-scale ranging from 1 (never true) to 5 (very often true). Items are totaled to create an overall maltreatment score. The abuse and neglect composite scores were calculated as the mean of the items from the physical, sexual, and emotional abuse subscales and the emotional and physical neglect subscales, respectively [54]. Possible scores range from 15 to 75 on the abuse subscale and 10 to 50 on the neglect subscale. Both subscales in the current sample demonstrated high reliability (neglect: Cronbach’s α = 0.88; abuse: Cronbach’s α = 0.91) as did the overall measure (Cronbach’s α = 0.93).

#### Anxiety and depressive symptoms

Symptoms of anxiety and depression were measured using the OASIS [45] and PHQ-9 [46], respectively (see Supplement).

### Region of interest analyses

Image acquisition and preprocessing are detailed in the Supplement. To evaluate whether childhood adversity predicted striatal activation during the anticipation of receiving social reward or avoiding social punishment, we examined associations between childhood neglect and abuse and neural activation in a priori regions of interest (ROI). ROIs were defined using an open-access anatomical mask of the striatum (Harvard-Oxford anatomical mask), which included the caudate, putamen, and VS. Beta coefficients reflecting voxel-wise activation were extracted for and averaged within each region and across left and right hemispheres for each participant, such that there were 3 ROIs considered in analyses. We restricted our analyses to focus on high incentive anticipation trials as these trials have demonstrated the best reliability in eliciting activation in the striatum [55]. Additionally, due to the short fixed interval (2000ms) between phases in the SID task, we were unable to clearly separate BOLD signal during anticipation phases from consumption phases. As such, only anticipation trials were examined.

We first examined bivariate correlations between abuse, neglect, depression and anxiety symptoms, and potential confounding demographic variables including age, gender, race, and ethnicity (see Figure S2 in the Supplement). Next, linear regression analyses were performed separately by type of incentive (i.e., reward, punishment) with childhood abuse or neglect as the independent variable and striatal response in the VS, caudate, or putamen as the dependent variable, resulting in 12 separate regression models. Sample size was calculated to ensure adequate power to detect small to medium effect sizes. All models were adjusted for any significant demographic or symptom covariates identified by the bivariate correlations. Multivariate outliers were screened using Cook’s distance (>1) and standardized residuals (±3) [56]. Outliers were detected for 3 regression models, thus, respective analyses were run with and without outliers included. To correct for multiple comparisons, we utilized FDR-correction within each incentive type (i.e., 6 comparisons each for reward and punishment). Finally, to explore unique effects of childhood abuse or neglect on striatal response, we conducted 6 regression models (i.e., 3 models for each incentive type) that included both types of adversity within the same model. Analyses were conducted in IBM SPSS, version 29 (analysis code available upon request).

## Results

### Relationships among childhood adversity, depression and anxiety symptoms, and demographic variables

Correlational analyses revealed a significant negative association between childhood abuse severity (*M* = 25.74, *SD* = 10.91, Range = 15–58) and childhood neglect severity (*M* = 29.05, *SD* = 5.65, Range = 15–38) (*r* = −0.58, *p* < 0.001, Supplemental Figure S2).^1^

There were no other significant associations between demographic factors (gender, age, race, ethnicity) and childhood abuse or neglect. Depression and anxiety symptoms were significantly correlated with childhood abuse (*r* = 0.28, *p* = 0.034; *r* = 0.26, *p* = 0.049, respectively), but not childhood neglect (*r* = −0.07, *p* = 0.622; *r* = −0.08, *p* = 0.540, respectively; Supplemental Figure S2).

### Striatal activation during anticipation of social reward

**Fig. 1:**
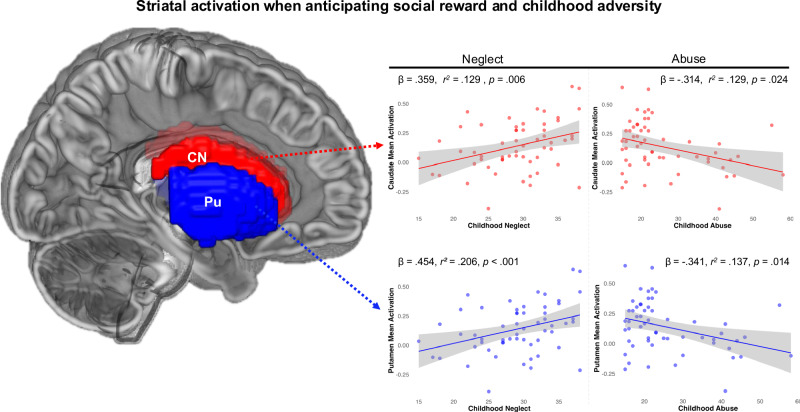
Associations between striatal activation when anticipating social reward and childhood adversity. *Note*. CN = caudate nucleus; Pu= putamen. Activation during the anticipation of social reward in these regions was differentially associated with childhood neglect and abuse. While childhood neglect predicted increased activation in dorsal regions, childhood abuse predicted reduced activation.

The SID task elicited robust activation in the striatum and other reward-related regions during the anticipation of social reward (see Supplement for results of task effect). Linear regression models predicting activation during the anticipation of social reward in striatal regions revealed that higher childhood neglect significantly predicted greater activation in the caudate (β = 0.359, *t* = 2.86, *p* = 0.006, *R*^*2*^ = 0.129, 95% CI = 0.004 to 0.023) and putamen (β = 0.454, *t* = 3.78, *p* < 0.001, *R²* = 0.206, 95% CI = 0.008 to 0.025) during the anticipation of social reward (Fig. 1). Childhood neglect did not significantly predict VS activation (β = 0.227, *t* = 1.73, *p* = 0.089, *R*^*2*^ = 0.052, 95% CI = −0.002 to 0.022).

Separate linear regression models revealed greater severity of childhood abuse significantly predicted decreased neural activation during the anticipation of social reward in the caudate (β = −0.314, *t* = −2.33, *p* = 0.024, *R*^*2*^ = 0.129 (overall model), 95% CI = −0.011 to −0.001) and putamen (β = −0.341, *t* = −2.54, *p* = 0.014, *R*^*2*^ = 0.137 (overall model), 95% CI = −0.011 to −0.001), after controlling for depression and anxiety symptoms (Fig. 1). Childhood abuse did not significantly predict activation in the VS (β = −0.244, *t* = −1.75, *p* = 0.086, *R*^*2*^ = 0.070 (overall model), 95% CI = −0.012 to 0.001). All findings survived FDR-correction.

Finally, regression models including both childhood neglect and abuse revealed a main effect of neglect, such that greater severity of childhood neglect predicted increased activation during the anticipation of social reward in the putamen (β = 0.402, *t* = 2.69, *p* = 0.009, *R*^*2*^ = 0.242 (overall model), 95% CI = 0.004 to 0.025), but not the ventral striatum (β = 0.124, *t* = 0.75, *p* = 455, *R*^*2*^ = 0.080 (overall model), 95% CI = −0.009 to 0.021) or caudate (β = 0.260, *t* = 1.66, *p* = 0.103, *R*^*2*^ = 0.173 (overall model), 95% CI = −0.002 to 0.021). There were no significant main effects of abuse (VS: β = −0.170, *t* = −0.97, *p* = 0.336, *R*^*2*^ = 0.080 (overall model), 95% CI = −0.012 to 0.004; caudate: β = −0.155, *t* = −0.94, *p* = 0.350, *R*^*2*^ = 0.173 (overall model), 95% CI = −0.009 to 0.003; putamen: β = −0.094, *t* = −0.60, *p* = 0.553, *R*^*2*^ = 0.242 (overall model), 95% CI = −0.008 to 0.004).

### Striatal activation during anticipation of avoiding social punishment

The task elicited robust activation in reward circuitry during the anticipation of avoiding social punishment (see Supplement for task effect). Linear regression models predicting activation during the anticipation of avoiding social punishment in striatal regions revealed childhood neglect was a significant predictor of activation in the putamen (β = 0.310, *t* = 2.41, *p* = 0.019, *R²* = 0.096, 95% CI = 0.002 to 0.020). Controlling for depression and anxiety symptoms, childhood abuse was also a significant predictor of activation in the putamen (β = −0.299, *t* = −2.16, *p* = 0.035, *R²* = 0.087, 95% CI = −0.010 to 0.000). However, neither of these findings survived FDR-correction. Neither childhood abuse or neglect significantly predicted activation in the VS (abuse: β = −0.180, *t* = −1.27, *p* = 0.209, *R²* = 0.049, 95% CI = −0.011 to 0.002; neglect: β = 0.054, *t* = 0.40, *p* = 0.690, *R²* = 0.003, 95% CI = −0.010 to 0.015) and caudate (abuse: β = −0.247, *t* = −1.80, *p* = 0.078, *R²* = 0.097, 95% CI = −0.010 to 0.001; neglect: β = 0.221, *t* = 1.68, *p* = 0.099, *R²* = 0.049, 95% CI = −0.002 to 0.017). Multivariate outliers were detected in 3 of the above models, but after removing outliers results of the analyses did not change.

In regression models including both childhood neglect and abuse, there were no significant main effects of abuse (VS: β = −0.234, *t* = −1.34, *p* = 0.188, *R*^*2*^ = 0.054 (overall model), 95% CI = −0.014 to 0.003; caudate: β = −0.185, *t* = −1.08, *p* = 0.284, *R*^*2*^ = 0.104 (overall model), 95% CI = −0.009 to 0.003; putamen: β = −0.169, *t* = −1.00, *p* = 0.324, *R*^*2*^ = 0.117 (overall model), 95% CI = −0.009 to 0.003) or neglect (VS: β = −0.089, *t* = −0.53, *p* = 0.598, *R*^*2*^ = 0.054 (overall model), 95% CI = −0.019 to 0.011; caudate: β = 0.102, *t* = 0.62, *p* = 0.535, *R*^*2*^ = 0.104 (overall model), 95% CI = −0.008 to 0.015; putamen: β = 0.212, *t* = 1.31, *p* = 0.195, *R*^*2*^ = 0.117 (overall model), 95% CI = −0.004 to 0.019) on striatal activation during the avoidance of social punishment.

## Discussion

In a transdiagnostic sample of adults characterized by social disconnection with clinically impairing depression or anxiety, we examined whether childhood abuse and neglect predicted striatal activation during the anticipation of social reward or avoidance of social punishment. The findings partially supported our hypotheses-- childhood abuse and neglect were differentially associated with dorsal striatal activation during the anticipation of social reward. However, when both neglect and abuse were included in the same model, only neglect remained a significant predictor of this activation. We did not observe any associations between either adversity type and activation in the VS. Additionally, childhood adversity did not significantly predict striatal activation while anticipating social punishment, though the magnitude and direction of effects were similar to those during anticipation of social reward. To our knowledge, this study was the first to explore how overall childhood neglect and abuse affect social reward processing in adults using an fMRI task probing social approach behaviors. In sum, we extend the current literature on early adversity, social reward processing, and psychopathology later in life, and suggest differing neural consequences of childhood abuse and neglect into adulthood.

As hypothesized, we observed associations between childhood abuse and neglect and striatal activation during the anticipation of social reward. These associations were observed in dorsal striatal regions but not the VS. Meta-analytic findings demonstrate that the striatum reliably activates during anticipatory phases of social reward [37], though activation in subregions likely reflect distinct aspects of reward processing. While the VS is involved in valuation of potential reward and incentive motivation, dorsal regions (caudate, putamen) trigger approach behavior [17, 42]. One possible interpretation, though speculative, is that individuals who experienced abuse or neglect may similarly evaluate potential social reward but differ in social approach behavior. While associations between childhood adversity and VS activation are well-replicated findings in the broader adversity literature (e.g., 5,18,22), few studies have considered *social* reward processing. Of the studies exploring social reward, those that found childhood abuse or neglect to predict VS activation used passive viewing tasks [32, 34]. Further, activation during these tasks was observed during the consumption phase of reward, not anticipatory phases wherein approach behaviors are enacted to obtain reward. Although Seitz and colleagues [36] observed associations between maternal antipathy and reduced VS activation during the anticipation of social reward, they did not observe associations with sexual abuse or maternal or paternal abuse or neglect (assessed via 5 separate subscales). Thus, it remains unclear whether VS function during anticipation of social reward is impacted by overall experiences of childhood abuse and neglect. Additional work is needed to explore potential associations during anticipatory phases of social reward processing.

In alignment with dimensional perspectives of early adversity [29], we observed that greater experiences of childhood neglect predicted increased activation in the dorsal striatum, while those with greater childhood abuse showed reduced activation. These two types of early adverse social experiences may impact individuals in different ways, resulting in multiple pathways towards social impairment in adulthood. However, given the negative correlation between abuse and neglect observed in the current sample, we also explored whether these adversity types had independent effects on striatal activation. When abuse and neglect were modeled together, neglect remained a significant predictor of putamen activation during anticipation of social reward, whereas abuse did not. This pattern suggests that in the current sample, neglect may exert a more direct and robust influence on social reward processes, while the effects of abuse observed may reflect shared variance with neglect or operate through indirect pathways. This may be a result of the notably more severe levels of neglect reported relative to abuse (near the threshold for severe emotional and physical neglect [57]; and above scores observed in other similar clinical samples [58, 59]), which may have limited our ability to detect unique effects of abuse in the shared model. Future research should examine whether these findings generalize to larger samples with more typical distributions of maltreatment or to individuals experiencing high levels of both abuse and neglect simultaneously, which may allow us to better disentangle the unique impacts of abuse and neglect on social reward processes. Additional individual factors surrounding childhood adversity, including developmental timing, predictability, and chronicity of adverse experiences [60] may also influence neural processing of social reward and punishment, and should be examined in future work alongside dimensions of abuse and neglect.

When considering the long-term impact of neglect on social functioning, a lack of positive social connections in a socially deprived childhood environment could lead to a heightened sensitivity to potential social rewards. As social reward in these environments is limited, heightened activation may reflect compensatory mechanisms to maximize the chance of receiving social reward when available. In adults, greater levels of loneliness are associated with heightened striatal activation to images of their close others, which may reflect a “social craving” response from the brain that mirrors craving responses individuals demonstrate to food when they are hungry [61, 62]. Perhaps participants with greater childhood neglect experience greater “social craving” in adulthood, resulting in increased approach behaviors for social engagement relative to those who were not deprived of important early social connections.

In contrast, we observed that greater abuse was associated with decreased striatal activation to social reward, though this finding was not observed when abuse and neglect were included as covariates in the same model. For individuals who repeatedly experienced harm when they may be expecting reward from social interactions in childhood, positive social stimuli may signal threat rather than reward. Therefore, a blunted striatal response may reflect downregulation of the reward system to avoid enacting approach behaviors. This interpretation aligns with prior work that found children who experienced harsh and/or abusive parenting were more likely to interpret positive interactions as negative [63]. Similar findings were observed in adults with social anxiety disorder [10]. Alternatively, blunted activation to social reward may reflect anhedonic behavior, where positive social interactions may be less rewarding, and thus less worthy of approach towards social reward. Theoretical models have linked stress, particularly social stress, to anhedonic behavior via dysfunction in the reward system [7, 64]. In this case, stressful childhood experiences of abuse may result in long-lasting dysfunction in social reward processing, ultimately presenting in anhedonic neural responses (i.e., blunted striatal response to social reward).

Meta-analytic work using the SID task suggests that the striatum reliably activates during anticipation of both reward and punishment [37]. Our exploratory analyses did not detect significant associations between childhood abuse and neglect with striatal activation during anticipation of social punishment. Although not statistically significant, we noted small to medium effect sizes between childhood abuse and neglect and activation in dorsal striatal regions (i.e., putamen) when anticipating avoiding social punishment, with the magnitude and direction of effects being similar to associations with activation during anticipation of social reward. There are several factors that may explain the absence of significant punishment-related findings. First, our task effect analyses revealed greater activation in reward regions during anticipation of avoiding punishment compared to obtaining reward, suggesting the opportunity to avoid social punishment may be particularly salient in this sample. This strong and potentially uniform response to punishment cues may have obscured individual differences related to childhood adversity. Indeed, avoiding social punishment may be perceived as highly rewarding regardless of early life experiences, particularly among people with clinically impairing anxiety or depression and social impairment. It is also possible that punishment processing is genuinely less affected by early adversity than reward processing, or that our modest sample size limited statistical power to detect more subtle associations. While our findings tentatively suggest childhood adversity may predispose individuals to similar neural responses to both potential social reward and threat, replication in larger samples is needed.

The current study had several notable strengths, including the exploration of social reward via an incentive delay task (cf. passive viewing of social stimuli) to capture neural mechanisms believed to underlie anticipatory social approach behavior, contributing to a limited literature in this area. Further, our data are from a transdiagnostic adult sample across a wide age range characterized by social impairment, an understudied population in the early adversity literature. However, results should be considered within the context of the following limitations. Our study design was cross-sectional and leveraged retrospective self-reported childhood adversity data, which may not fully align with prospective reports or external documentation of adversity (e.g., legal records) [65]. Nevertheless, there may be pragmatic reasons to use retrospective reports of early adversity in adult clinical settings (e.g., ease of administration, unavailability of corroborating reports), and the current findings point to ecophenotypes that could be clinically actionable [66]. Future work exploring associations between childhood adversity and neural responses to social reward with longitudinal and corroborating data is needed. Moreover, it cannot be determined whether these associations are the direct result of experiencing early adversity, or if they instead reflect the interaction of a history of early adversity and current internalizing symptoms. Future work should consider including an adversity-exposed group of individuals without psychiatric diagnoses to better determine the unique contributions of early adversity on the neural mechanisms of social reward processing. Our task design also limited our ability to explore activation during both anticipation and consumption phases, restricting our ability to compare how childhood adversity impacts multiple phases of reward processing within a single sample. Finally, our analytic approach was intentionally confined to a priori regions in the striatum to limit comparisons and increase statistical power to detect effects given the exploratory nature of the study and modest sample size. This small size limits both the robustness and generalizability of our findings. Broader networks involved in social reward processing (e.g., prefrontal regions, insula) may also be related to different dimensions of childhood adversity and should be explored in larger samples.

Our study provides evidence that childhood abuse and neglect may produce distinct effects on social reward processing that may persist into adulthood. Within a clinical sample of adults experiencing depression and/or anxiety and social functioning impairments, the type of childhood adversity experienced contributed to individual differences in social reward processing within the same brain regions. Thus, our findings support a dimensional model of early adversity and build on a larger theoretical framework contending that even within psychological disorders, individuals with childhood adversity may display distinct neurobiological profiles that differ from those who have not experienced adversity [66]. While further work is needed, particularly with longitudinal data, our study provides a foundation for future investigation into how treatment approaches could be tailored to address impairments specifically linked to different types of childhood adversity.

## Supplementary information

Supplemental Material

## Data Availability

Deidentified data, analysis code, and research materials are available on request from the authors.

## Footnotes

1 2 outliers (>1.5 times the interquartile range) were observed in the threat subscale. When these were removed, the correlation remained significant and of a similar strength and direction, (r = −0.53, *p* < 0.001). Additionally, a Spearman’s correlation was run to account for the skewed distributions of the subscales, which resulted in a similar association (r_s_ = −0.60, *p* < 0.001).
