# Supplementary material for: Differential associations of childhood abuse and neglect with neural responses to social reward and punishment in adults with anxiety or depression
Source: Transl Psychiatry. 2026 Feb 6;16:86. doi: 10.1038/s41398-026-03881-2 (PMC12923754; doi:10.1038/s41398-026-03881-2)
Supplement: Supplementary file 1 — Supplemental Material [file 41398_2026_3881_MOESM1_ESM.docx]

**SUPPLEMENTARY INFORMATION**

**Differential associations of childhood abuse and neglect with neural responses to social reward and punishment in adults with anxiety or depression**

Spaulding et al.

**Methods**

**Participants**

Exclusion criteria included 1) active suicidal ideation with intent; 2) moderate to severe alcohol or marijuana use disorder in the past year or mild to severe substance use disorder (all other drugs); 3) bipolar I or psychotic disorders; 4) moderate to severe traumatic brain injury, history of a major neurological disorder, or severe or unstable medical conditions, 5) concurrent psychotropic medication use; 6) concurrent psychotherapy (unless 12-week stability criteria had been met); and 7) magnetic resonance imaging (MRI) contraindications.

**Social Incentive Delay Task**

In the social incentive delay (SID) task, participants had the opportunity to gain social reward or avoid social punishment. Participants were presented with a cue shape indicating the level of potential reward or punishment (i.e., low, high). Smiling facial expressions were used as social reward, angry facial expressions were used as punishment. Facial expressions varied in intensity to reflect the level of feedback (e.g., closed smile for low reward, grin for high reward). Participants could receive social reward or avoid punishment through successful on-time response to the target. During reward blocks, a neutral outcome of a blurred facial control stimulus was shown if participants did not respond quickly enough. During punishment blocks, this neutral stimulus was shown if participants responded on-time to the target, otherwise an angry facial expression was presented. Participants completed 2 runs of the task. In each run, reward and punishment were presented in separate blocks for a total of 54 trials per run that were counterbalanced across participants. Each trial included a presentation of the cue (250ms), a delay period (2250-2750, jittered), target symbol (250ms at the start of the task and adjusted throughout task depending on participant performance), anticipation phase (300ms), receipt of reward, punishment, or neutral cue (1650ms). See Figure S1 for visualization of the SID task.

**Measures**

***Anxiety and Depressive Symptoms***

The OASIS was used to measure the frequency and severity of anxiety symptoms over the two previous weeks and consists of 5 items rated on a 0 (never/none/not at all) to 4 (constantly/extreme/all the time) scale (Norman et al., 2006). Depressive symptoms were measured using the PHQ-9 (Kroenke et al., 2001). This scale consists of 9 items rated on a 0 (not at all) to 3 (nearly every day) scale and measures self-reported severity of symptoms of depression over the last two weeks. The current sample’s average OASIS (*M*=10.65, *SD*=2.97) and PHQ-9 (*M*=12.25, *SD*=4.91) scores indicate moderate levels of anxiety and depressive symptoms, respectively.

**Image Acquisition**

Participants were scanned using a 3T General Electric 750 scanner with a 8-channel head coil. T2* blood oxygen level dependent (BOLD) images were acquired across 2 runs using echo-planar imaging (EPI) scans (voxel size = 3.5; slice thickness=3mm, slice spacing=1mm, TR=1.5s, TE=32ms, matrix=64 x 64, FOV=240mm, flip angle=80ﹾ, axial plane). Anatomical T1-weighted images were acquired using spoiled gradient recalled (SPGR) sequence (172 slices, thickness=1mm; TI=450ms, TR=8ms, TE=3ms, matrix size= 192X256; FOV=256 cm; flip angle=12ﹾ; sagittal plane).

**Image Preprocessing and Analysis**

Preprocessing protocols were conducted using Analysis of Functional NeuroImages (AFNI; <https://afni.nimh.nih.gov/afni>). Preprocessing steps within the afni proc.py tool included despiking, slice-time correction, functional image realignment, co-registration of anatomical and functional scans, spatial smoothing of 6mm, and non-linear spatial standardization to MNI space. Subjects with poor data quality were excluded (n=2): 1 participant did not complete both runs of the SID (i.e., 50% missing data), 1 participant had low task engagement (i.e., <20% accuracy rate on reward trials). For each individual subject, preprocessed time series data were analyzed in a multiple regression model including motion and task regressors. Anticipation and consumption trials were separately coded into regressors of interest modeling levels of incentive (none, low, high) and type of incentive (reward, punishment). Consumption trials also included a regressor representing performance (hit, miss). Regressors were shifted by a gammavariate hemodynamic response function (AFNI:waver) and then entered into a generalized linear model to calculate beta coefficients.

**Whole Brain Analysis**

To explore the main effect of reward or punishment during anticipation trials in the SID task, we conducted a whole brain analysis via AFNI’s 3dLME program. Our contrast of interest was the reward vs. no reward contrast (or punishment vs. no punishment). We applied a conservative voxel-wise threshold of p < 0.001. Cluster threshold was calculated using AFNI’s 3dClustSim with the mixed-model spatial autocorrelation function (-acf) and the NN1 bisided option. This resulted in a whole-brain corrected cluster threshold of *k* = 49, *p* < .05.

**Results**

**Task Effect of the SID**

Results of the whole brain analysis revealed increased activation in reward circuitry during the anticipation of social reward. The SID task elicited robust activation in reward regions, including the caudate nucleus, putamen, and thalamus. Additionally, activation was observed in the motor and visual cortices (see Figure S4). Activation was observed in similar regions during the anticipation of avoiding social punishment (see Figure S5).

**Figure S1.** *Experimental paradigm for the Social Incentive Delay (SID) task, modified after Spreckelmeyer et al. (2009).*

**
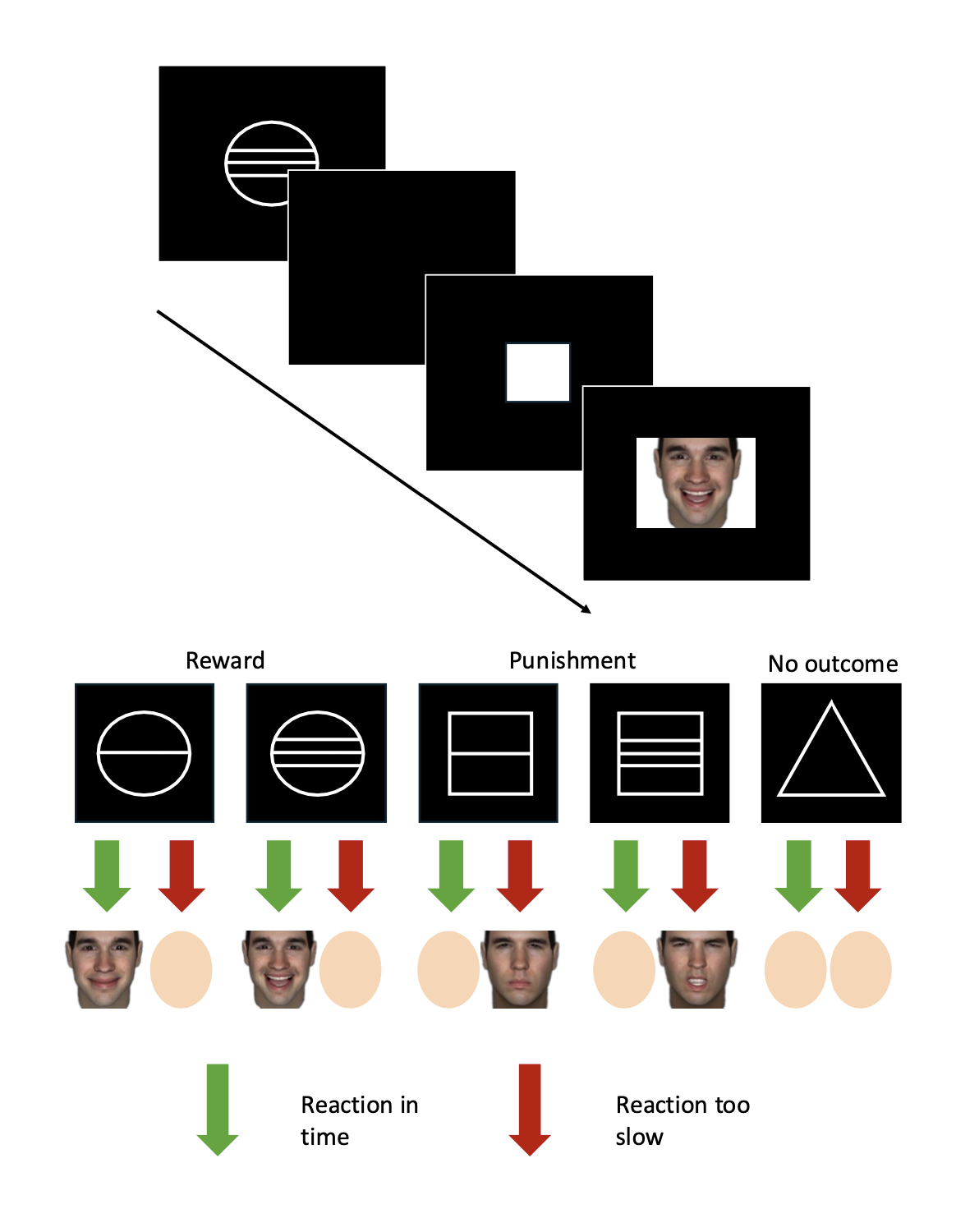
**

**Figure S2.** *(A) Correlation Matrix including childhood abuse, childhood neglect, depression symptoms (PHQ-9), and anxiety symptoms (OASIS). (B) Scatterplot showing the relationship between childhood abuse and neglect.*


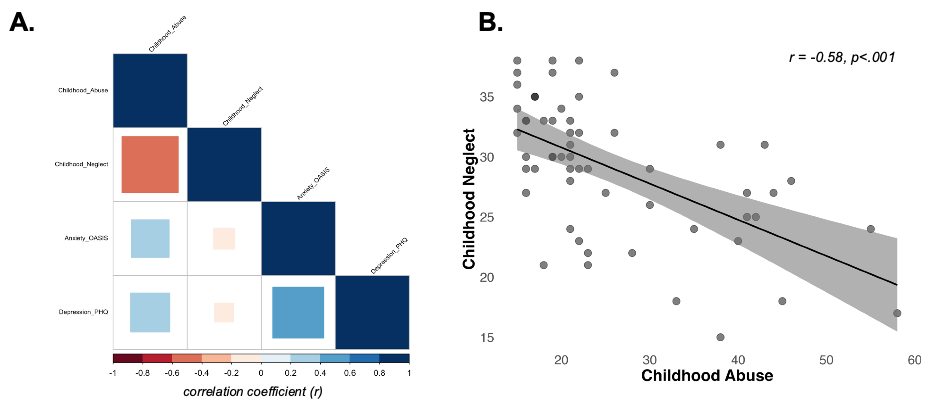


**Figure S3.** *(A) Frequency and density of childhood neglect. (B) Frequency and density of childhood abuse.*

**Figure S4.** *Task effects of the Social Incentive Delay Task during anticipation of social reward in the present sample. Brain image represent coronal sections (left=left) with height threshold set at p<.001.*

***
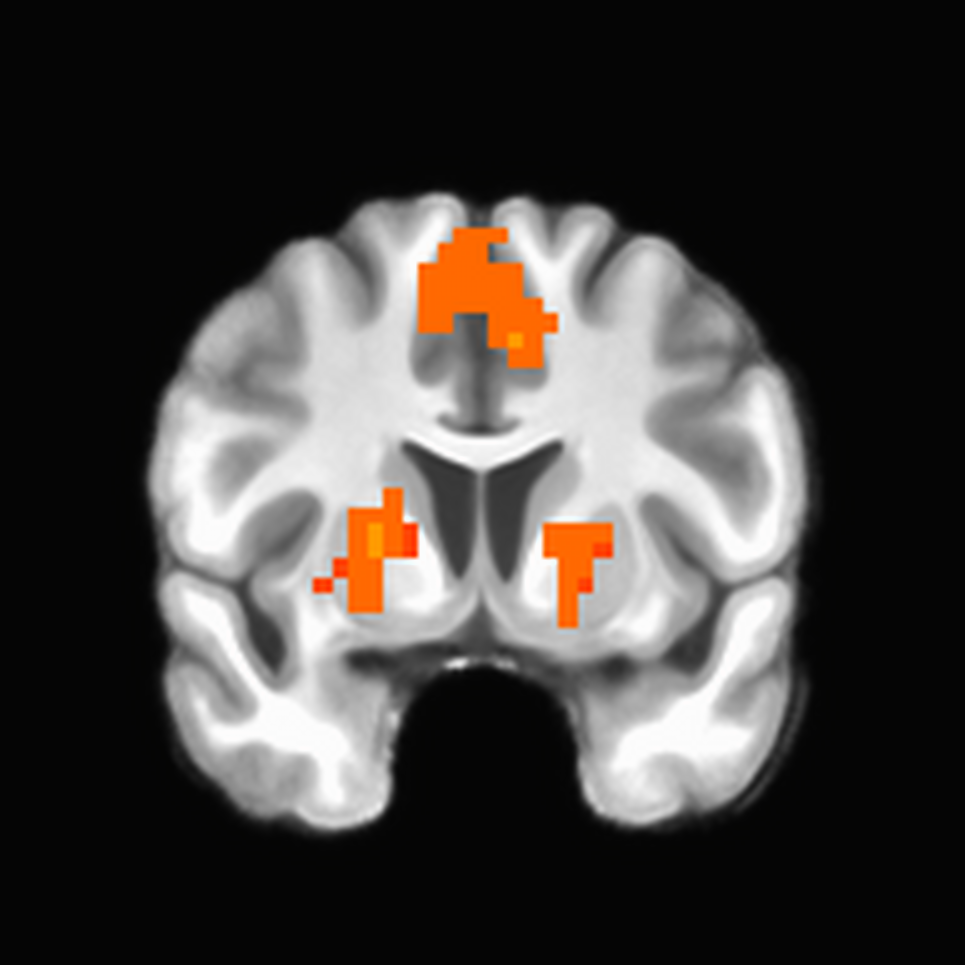
***

**Figure S5.** *Task effects of the Social Incentive Delay Task during anticipation of avoiding social punishment in the present sample. Brain image represent coronal sections (left=left) with height threshold set at p<.001.*

***
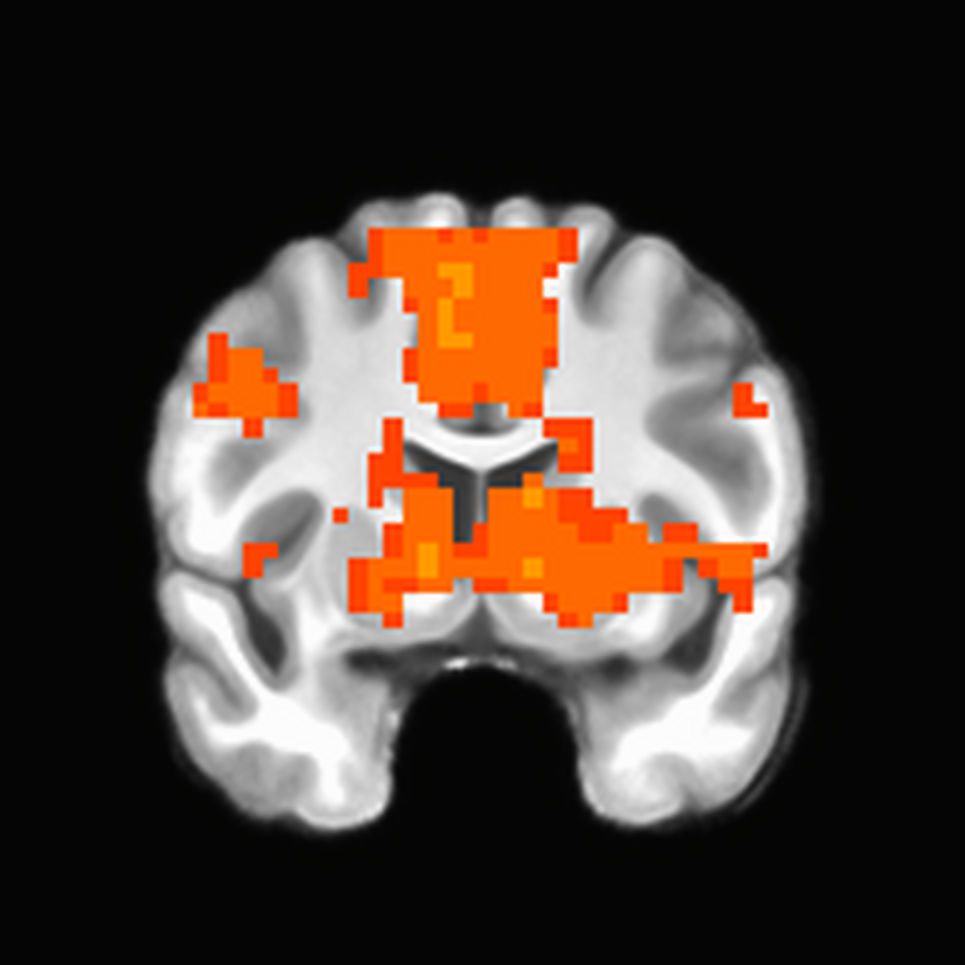
***

**References**

1. Norman, S. B., Hami Cissell, S., Means-Christensen, A. J., & Stein, M. B. (2006). Development and validation of an Overall Anxiety Severity And Impairment Scale (OASIS). *Depression and Anxiety, 23*(4), 245–249.<https://doi.org/10.1002/da.20182>
2. Kroenke, K., Spitzer, R. L., & Williams, J. B. W. (2001). The PHQ-9. *Journal of General Internal Medicine, 16*(9), 606–613.<https://doi.org/10.1046/j.1525-1497.2001.016009606.x>
3. Spreckelmeyer, K. N., Krach, S., Kohls, G., Rademacher, L., Irmak, A., Konrad, K., Kircher, T., & Gründer, G. (2009). Anticipation of monetary and social reward differently activates mesolimbic brain structures in men and women. *Social Cognitive and Affective Neuroscience*, *4*(2), 158–165.<https://doi.org/10.1093/scan/nsn051>
